# A Novel Model to Combine Clinical and Pathway-Based Transcriptomic Information for the Prognosis Prediction of Breast Cancer

**DOI:** 10.1371/journal.pcbi.1003851

**Published:** 2014-09-18

**Authors:** Sijia Huang, Cameron Yee, Travers Ching, Herbert Yu, Lana X. Garmire

**Affiliations:** 1 Molecular Biosciences and Bioengineering Graduate Program, University of Hawaii at Manoa, Honolulu, Hawaii, United States of America; 2 Epidemiology Program, University of Hawaii Cancer Center, Honolulu, Hawaii, United States of America; 3 Neurobiology Program of Biology Department, University of Washington, Seattle, Washington, United States of America; University of Chicago, United States of America

## Abstract

Breast cancer is the most common malignancy in women worldwide. With the increasing awareness of heterogeneity in breast cancers, better prediction of breast cancer prognosis is much needed for more personalized treatment and disease management. Towards this goal, we have developed a novel computational model for breast cancer prognosis by combining the Pathway Deregulation Score (PDS) based pathifier algorithm, Cox regression and L1-LASSO penalization method. We trained the model on a set of 236 patients with gene expression data and clinical information, and validated the performance on three diversified testing data sets of 606 patients. To evaluate the performance of the model, we conducted survival analysis of the dichotomized groups, and compared the areas under the curve based on the binary classification. The resulting prognosis genomic model is composed of fifteen pathways (e.g. P53 pathway) that had previously reported cancer relevance, and it successfully differentiated relapse in the training set (log rank p-value = 6.25e-12) and three testing data sets (log rank p-value<0.0005). Moreover, the pathway-based genomic models consistently performed better than gene-based models on all four data sets. We also find strong evidence that combining genomic information with clinical information improved the p-values of prognosis prediction by at least three orders of magnitude in comparison to using either genomic or clinical information alone. In summary, we propose a novel prognosis model that harnesses the pathway-based dysregulation as well as valuable clinical information. The selected pathways in our prognosis model are promising targets for therapeutic intervention.

## Author Summary

With the increasing awareness of heterogeneity in breast cancers, better prediction of breast cancer prognosis is much needed early on for more personalized treatment and management. Towards this goal we propose in this study a novel pathway-based prognosis prediction model, which emphasizes on individualized pathway-based risk measurement using the pathway dysregulation score (PDS). In combination with the L1-LASSO penalized feature selection and the COX-Proportional Hazards regression model, we have identified fifteen cancer relevant pathways using the pathway-based genomic model that successfully differentiated the relapse in the training set as well as three diversified test sets. Moreover, given the debate whether higher-order representative features, such as GO sets, pathways and network modules are superior to the gene-level features in the genomic models, we demonstrate that pathway-based genomic models consistently performed better than gene-based models in all four data sets. Last but not least, we show strong evidence that models that combine genomic information with clinical information improves the prognosis prediction significantly, in comparison to models that use either genomic or clinical information alone.

## Introduction

Breast cancer is the second (after skin cancer) most frequently diagnosed cancer in women, and ranks second (after lung cancer) in the deaths of women in year 2013 [1]. Most clinical studies categorize breast cancer into four molecular subtypes: Luminal A, Luminal B, Triple Negative/Basal like and Her2 [2], [3]. The survival outcomes differ significantly among the clinical subtypes. Luminal A and B subtypes have a relatively good prognosis, whereas triple negative or basal like tumors, and Her2 tumors have very poor prognosis with much higher recurrence and metastasis rates [2]–[4]. Furthermore, it is increasingly being realized that breast cancers are much more heterogeneous diseases than what is determined by the clinical subtypes, and that better prediction of prognosis is needed early on for more personalized treatment and management. Towards this goal, prognosis biomarkers of breast cancers have been investigated in many studies [5]–[7], based on signatures from high-throughput platforms such as gene expression profiles. Some signature panels such as the NKI 70 test are currently in commercial use with decent prediction of metastasis [8].

However, transcriptomic data are usually poorly dimensioned with many more genes than the number of samples, thus methods that reduce the dimension by incorporating higher-order information of functional units, such as gene sets, pathways and network modules, have been recently explored [9]–[16]. This methodology is based on the observation that multiple genes involved in the same biological processes are often dysfunctional all together in cancers [17], therefore features selected from representative functional units are presumably more robust with better biological annotations [10],[17]. Currently, two main approaches to define functional units have been proposed. One approach is to identify *de novo* functional units from the data. For example, van Vliet used an unsupervised module discovery method to identify gene modules, scored them and use them as features in a Bayes classifier [18]. Teschendorff et al. reported improved prognostic classification of breast cancers via a novel strategy to discover the activated pathways from the modules of “expression relevance network” [12]. Similarly, network analysis with combination of all the useful gene information has been developed and utilized to measure the coordination among the genes [13]. The other main approach uses the existing pathway information to build functional units. For example, Lee et al used the MsigDB C2 gene sets to select feature sets using the t-test, and represented the pathway activity level by a subset of genes whose combined expression delivered optimal discriminative power for the disease phenotype [14]. Abraham et. al used a set statistic that aggregated the expression levels of all genes in a set, and constructed prognostic gene sets that were as predictive as individual genes, yet more stable and interpretable within the biological context [9].

However, most of these methods model the prognosis as binary outcomes, and *post hoc* analyze the performance of the methods using survival information; or individualized information of pathway deregulation is lost during information extraction before deriving statistical metrics. More importantly, the merits of combining clinical features and genomic features together have not been adequately addressed in most studies, where the models were only built upon the genomic information. In this study, we use a novel pathway-based deregulation scoring matrix to transform the gene-based genomic features in combination with the Cox regression and L1-LASSO regularization to model survivals. With this pathway deregulation score matrix as inputs, we constructed a pathway-based genomic model consisting of fifteen cancer relevant pathways that successfully predicted relapse difference (log rank p-value = 6.25e-12, and AUC = 0.80) and validated them on three breast cancer data sets with diversified clinical profiles (log rank p-value<0.0005, and average AUC = 0.68). The pathway-based genomic models consistently performed better than gene-based models on all four data sets. Moreover, combining genomic level information with clinical information improved prognosis prediction and classification by at least three orders of magnitudes of p-values, in comparison to either genomic or clinical information alone.

## Results

### Data Summary

We used four individual gene expression microarray data sets for the testing and validation of the pathway-based prognosis model (Table 1), all of which were measured by Affymetrix HG-U133A array and had relapse and survival information. We used the data set of 236 patients in Miller et. al. [19] as the training data mainly because this data set contains the most abundant clinical information, including ER status, PG status, tumor size, grade, lymph node status and P53 mutation.

**Table 1:** Summary of patient and tumor characteristics.

Characteristics	Training Miller LD	Testing Set1 Pawitan Y	Testing Set2 Ivshina AV	Testing Set3 Desmedt D
No. of patients				
	236	159	249	198
Relapse, No. (%)				
Relapse	55 (23%)	40 (25%)	89 (35%)	91 (46%)
Non-relapse	181 (77%)	119 (75%)	160 (64%)	107 (54%)
Mean Relapse Free Survival (y)				
	8.167	5.959	7.142	9.312
Mean Age (year)				
	62.51		62.12	46.39
ER status, No. (%)				
Positive	201 (85%)		211 (85%)	134 (67%)
Negative	31 (13%)		34 (13%)	64 (33%)
NA	4 (2%)		4 (2%)	0
PG status, No. (%)				
Positive	57 (24%)			
Negative	179 (76%)			
NA	0			
Tumor Size(mm)				
<10 (*T*_1*a*_, *T*_1*b*_)	13 (6%)		14 (6%)	9 (4%)
10–20 (*T*_1*c*_)	92 (40%)		95 (38%)	59 (30%)
20–50 (*T*_2_)	123 (52%)		129 (52%)	129 (65%)
>50 (*T*_3_)	5 (2%)		10 (4%)	1 (1%)
Grade, No. (%)				
1	62 (26%)	28 (18%)	68 (27%)	30 (15%)
2	121 (51%)	58 (36%)	126 (51%)	83 (42%)
3	51 (22%)	61 (38%)	55 (22%)	83 (42%)
NA	2 (1%)	12 (8%)	0	2 (1%)
Lymph Node Status, No. (%)				
Positive	78 (33%)		81 (32%)	
Negative	149 (63%)		159 (64%)	198 (100%)
NA	9 (4%)		9 (4%)	
P53 Mutation Status, No. (%)				
Mutated	55 (23%)		58 (23%)	
Wild Type	181 (77%)		189 (76%)	
NA	0		2 (1%)	

**Figure 1:**
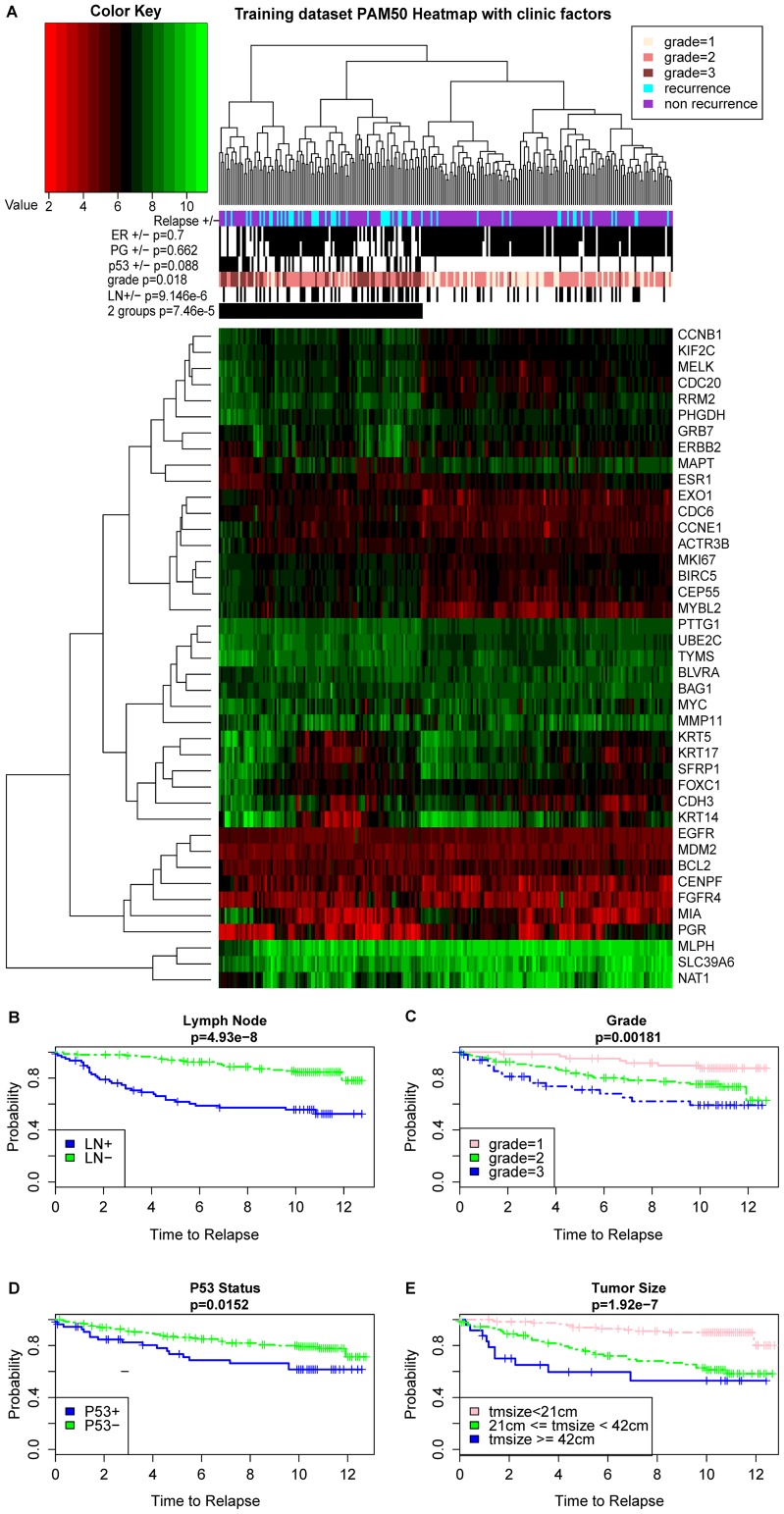
The PAM50 gene signatures and their association with clinical information in the training data set. A, The heatmap of the log2 transformed gene expression for PAM50 signatures. Green and red colors represent higher and lower expression levels, respectively. The samples are further categorized into two major groups based on the hierarchical clustering. The p-values of the clinical features such as ER, PG, P53, Grade, lymph node (LN) and dichotomized groups with relation to relapse status are calculated using Chi-square tests. B–E, Kaplan Meier survival estimates of relapse free survivals according to major clinical features: (B) Lymph node status, (C) Grade, (D) P53 mutation status and (E) Tumor size. P-values are calculated using Wilcoxon log-rank tests and (+) denotes the censored observations in the study.

PAM50 is a list of 50 genes initially proposed to successfully differentiate the breast cancer subtypes and it was later found that PAM50 also harbors good prognosis information on breast cancer [20]. Therefore, we first present the testing data summary results and correlate relapse with PAM50 and other clinical factors (Figure 1). Although tumor molecular subtypes are unknown due to the missing Her2 marker information, we nevertheless observed a good correlation between PAM50 matrix and relapse. Based on the hierarchical clustering results of PAM50 heatmap, we dichotomized the samples into high and low risk groups, This grouping approach, without any supervised learning, results in a fairly good association to relapse status (Chi-square test p = 7.46e-5). Additionally, grade and lymph node have significant associations to relapse, with Chi-square test p-values of 0.018 and 9.146e-6 respectively. Single clinical factor based survival analysis also confirms such significant relevance to relapse: p-values of Wilcoxon log rank tests for the p53, grade, tumor size and lymph node status based survival differences are 0.0152, 0.00181, 1.92e-7 and 4.93e-8, respectively. Similar to previous observations [21], ER and PG status are not good prognosis indicators, with the log rank test p-values of 0.819 and 0.227, respectively.

There are a total of around 600 samples in the three testing data sets, 2.5 times the size of samples in the training set. Testing set 1 (Ivshina data) [22] and testing set 2 (Pawitan data) [23] have very similar distribution pattern to the training data (Miller data) [19]. However testing set 3 (Desmedt data) [24] has very different distribution compared to other three data sets, as the samples were all lymph node negative tumors. We include set 3 as an extension to the other two testing data sets to exam the performance of the pathway-based genomic model for prognosis.

### Building the Pathway-Based Genomic Model

**Figure 2:**
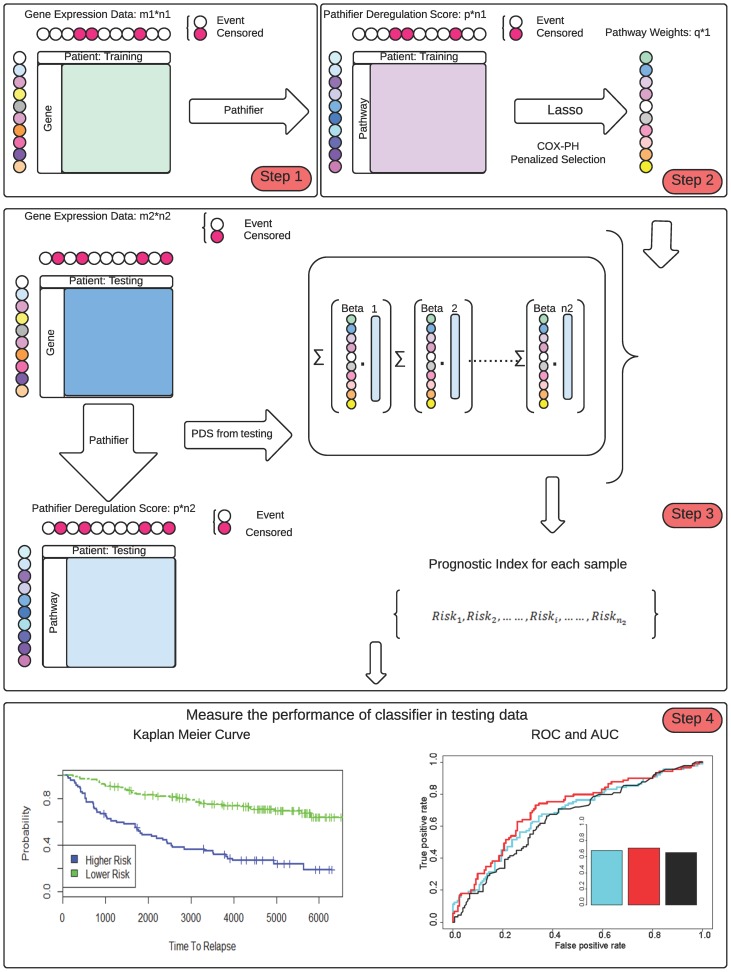
The workflow of the pathway-based genomic model. Step 1. Transform the input data in the training set: the gene-based expression data are transformed into the pathway-based data input through the *pathifier* algorithm, using the pathway information from KEGG and BIOCARTA. The new input matrix is represented by Pathway Deregulation Scores (PDS). Step 2. Build the prognosis prediction model. The PDS matrix is integrated with the survival information via a Cox-PH model under penalized feature selection using the L1- LASSO method. Featuring pathways are selected and the coefficients (or weights) of these pathways are estimated using log likelihood cross validation. Step 3. Set the relapse risk threshold from the model. The prognostic index (PI) cutoff value is determined from the model to match the ratio of relapse/non-relapse in the training set. This PI is used as the relapse risk threshold on all the testing sets where the sum of weighted PDS is calculated on the pathways selected in Step 2. The input PDS matrices of testing data sets are computed the same as in Step 1. Step 4. Evaluate the performance of the prognostic model. The performance is evaluated through Kaplan-Meier curves of the dichotomized risk groups by PI scores, as well as the ROC curves and AUC values.

We have developed a novel pathway-based prognosis prediction model, unlike most other models that are gene-based (Figure 2). We transformed a conventional gene-based matrix into a new pathway-based matrix of reduced numbers of rows, where each row represents a KEGG or BIOCARTA pathway-based scores over all samples (columns). Instead of using log2 transformed intensities as elements of the matrix, we used Pathway Dysregulation Scores (PDS) [25] that measure the distance of a particular pathway to the “normal condition” curve in a hyperspace. PDS ranges from 0 to 1, and the higher PDS score signifies more “abnormity”. This pathway-based PDS matrix was used as the initial input to select featuring pathways that are predictive of survival, based on the multi-variate Cox-PH model [26]. We used L1-LASSO penalization method [27]–[29] to constrain the featuring pathways to be selected. To be consistent, we conducted 250 simulations to select the best set of pathways.

**Figure 3:**
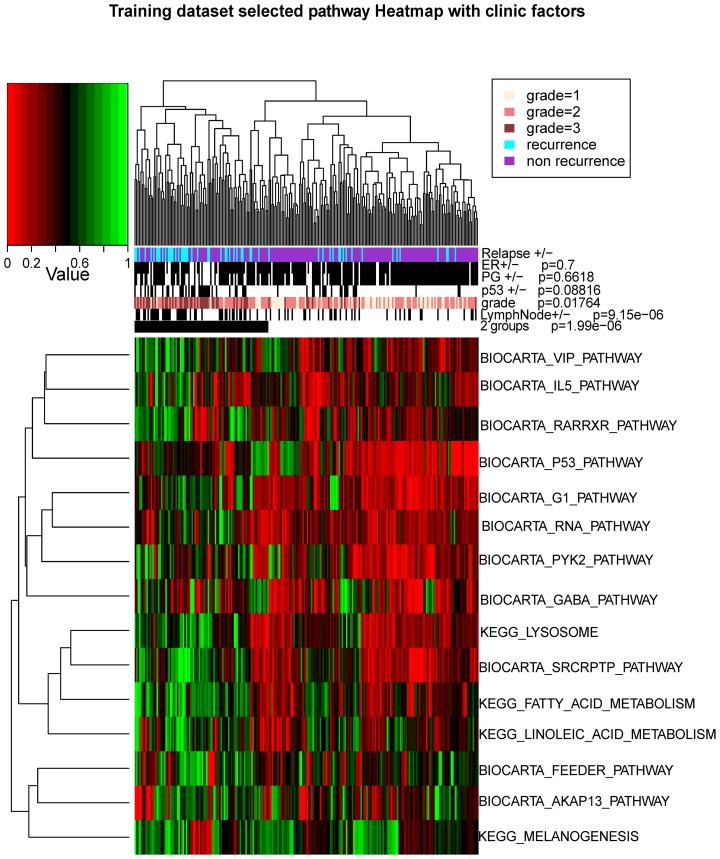
The selected pathway signatures and their association with clinical information in the training data set. The heatmap shows the patterns of Pathway Deregulation Score (PDS) of selected pathways in the genomic model. Green and red colors represent higher and lower PDS scores, respectively. The samples are further categorized into two major groups from hierarchical clustering, as in Figure 1. The p-values of the clinical features such as ER, PG, P53, Grade, lymph node (LN) and dichotomized groups with relation to relapse status are calculated using Chi-square tests.

We first evaluated the featuring pathways selected by the model, in relation to other clinical factors and relapse status in the training data set (Figure 3). Comparing the heatmap of selected featuring pathways to that of the PAM 50 genes (Figure 3A), the selected pathways are more prognostic for relapse. This is supported by two observations: (1) Dichotomized samples of high risk and low risk groups through hierarchical clustering of PDS scores have a higher correlation to relapse status (Chi-square test p = 1.99e-6), compared to those of PAM50 gene matrix (Chi-square test p = 7.46e-5) and (2) The median PDS scores over fifteen selected pathways have a correlation coefficient of 0.17 to relapse, in comparison to 0.08 for the median expression intensities over PAM50 genes. Thus the selected pathways by our model are better prognostic features than PAM50 genes, in terms of the correlation to disease relapse.

To investigate the performance of the model, we used the PI value which is the logarithm of hazard ratio from the fitted Cox-PH model to dichotomize the samples, similar to others [21] [30]. We divided the samples into higher and lower risk groups with a 3 to 1 ratio (3^rd^ quartile in PI), in order to match the relapse versus non-relapse sample ratio in the training data. Samples with larger PDS scores are expected to have higher PI scores, and are more likely to have relapsed diseases. The same PI threshold was applied to dichotomize the training data set as well as multiple independent testing data sets. The performance of the genomic model was then evaluated by two approaches: (1) the Wilcoxon log rank test p-values of the Kaplan-Meier survival curves from the two risk groups in each data set, and (2) the AUCs of ROC curve based on binary classification.

### The Pathway-Based Genomic Model Is Predictive on Multiple Testing Data Sets

**Figure 4:**
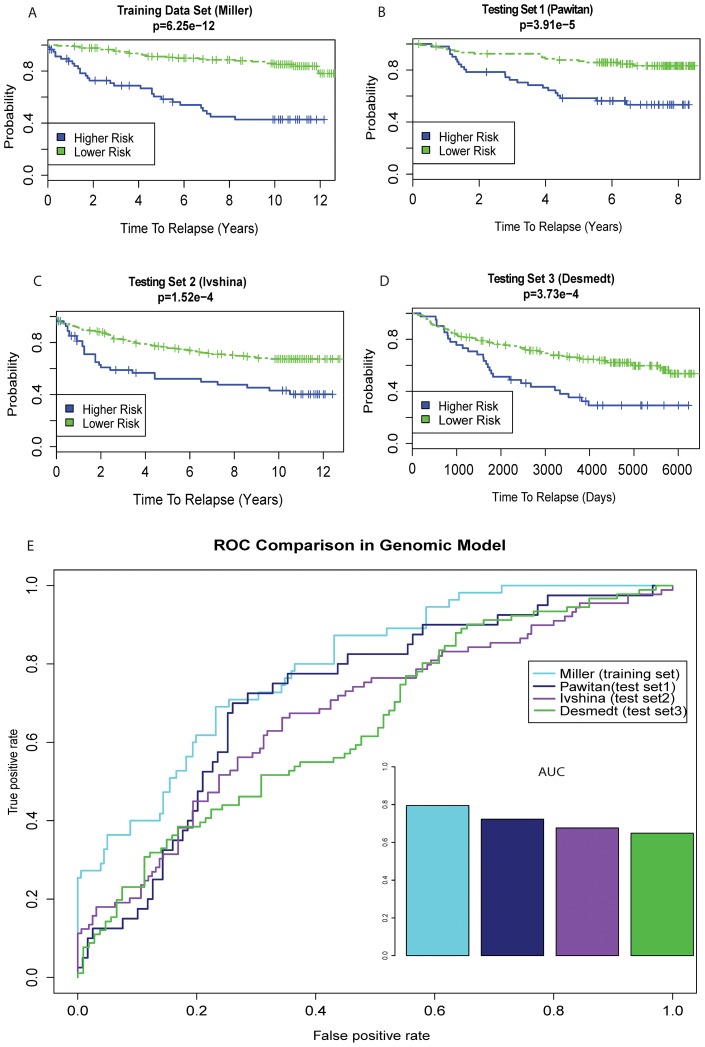
Prognosis performance of the pathway-based genomic model. A–D. A prognosis index (PI) is calculated from the training data set and applied to dichotomize samples in training (A) and testing data sets (B–D). Higher risk and lower risk groups determined by the PI cutoff are compared by Kaplan-Meier curves. P-values of the survival difference between the two groups are calculated using Wilcoxon log-rank tests and (+) denotes the censored observations in the study. E. ROC curves are generated using PI values as predictions in comparison to the relapse/non-relapse information. AUCs are listed as the insert.

Instead of combining all four data sets for meta-analysis, we kept them as individual data sets to validate the robustness of our model. As expected, the pathway-based genomic model is highly accurate at differentiating the risks of breast cancer relapse within the training data, with a Wilcoxon log rank p-value of 6.25e-12 (Figure 4A). The model yields very decent predictive results with the p-value of 1.52e-4 in testing set 1 and 3.91e-5 in testing set 2 (Figure 4B and 4C). The predictive performances are expected to drop in the testing data sets, since they have different patient populations and clinical characteristics from the training set (Table 1). Impressively, the model gives a very significant p-value of 3.73e-4 for testing data set 3 (Figure 4D), which are all early stage lymph node negative tumors whose prognosis is very difficult to predict. Additionally, we evaluated the performance of models using binary classification. We used the relapse/non-relapse information in the data sets as truth measures, and the model's high vs. low risk classification as predictions. As shown in Figure 4E, the ROC curve in the training set gives an AUC value of 0.80, and AUCs of 0.73 (testing set 1, Pawitan data), 0.67 (testing set 2, Ivshina data), 0.65 (testing set 3, Desmedt data), consistent with the results in Kaplan-Meier curves (Figure 4A–D).

To examine the effect of total number of input pathways on model performance, we randomly kept 1/2, 1/4, 1/8 and 1/16 of all input KEGG and BioCarta pathways in the training dataset, and then generated the PDS Matrices for 18 simulations under each scenario. For each simulation, we built the model with the same workflow as in Figure 2 and computed the Wilcoxon log-rank test p-value between the survival curves of the two risk groups, as well as the AUCs of the classification results. The boxplot in Figure S1 shows a gradual decrease of AUCs due to the input pathways, in the order of 1/2>1/4>1/8>1/16 pathway-based models. The difference between 1/2 and 1/4 pathways is significant (p-value<0.05). All AUCs, however, are in the range between 0.69 and 0.81.

### The Pathway-Based Genomic Model Is Superior to the Gene-Based Genomic Model

**Figure 5:**
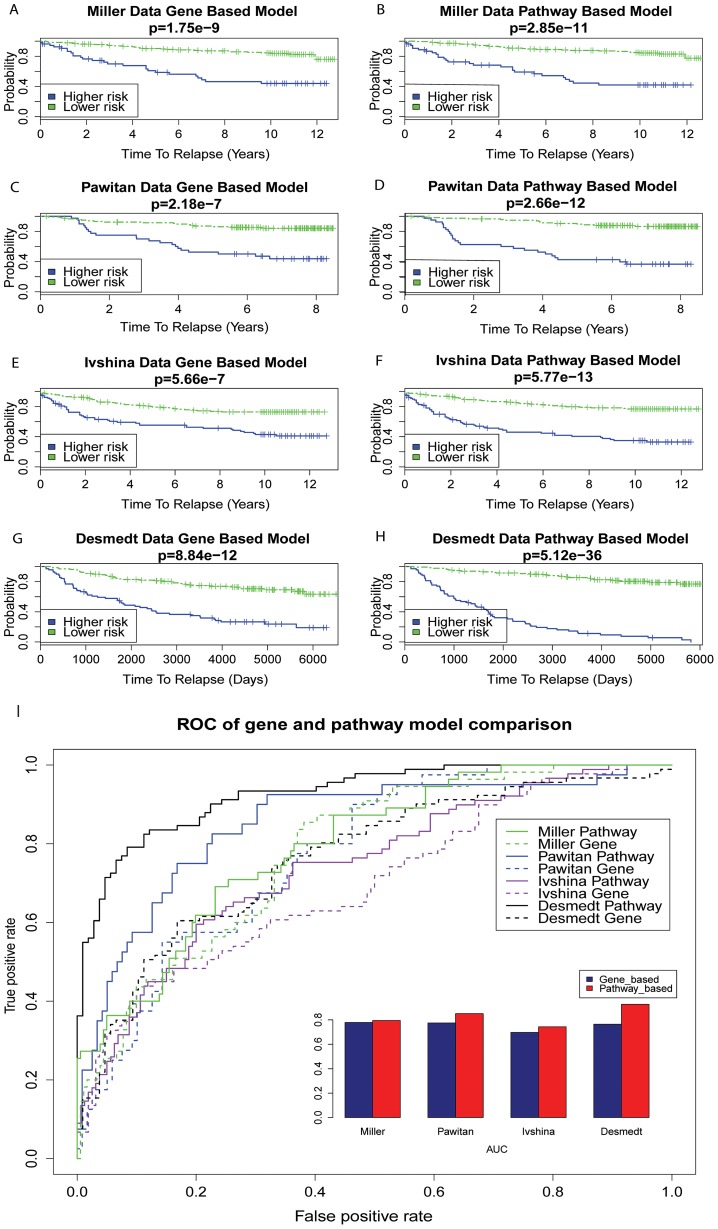
Comparing the prognosis performance between the gene-based and the pathway-based genomic models. A–H. Gene-based and pathway-based genomic models are trained individually on the data sets. The PI is calculated to match the ratio of relapse to non-relapse on each data set and used to dichotomize the samples into higher risk and lower risk groups, similar to Figure 4. The associated p-values in Kaplan Meier curves are calculated using the Wilcoxon log-rank tests, as in Figure 4. Pathway-based genomic models consistently outperform alternative gene-based genomic models in all data sets. I. ROC curves are generated from PI based classification predictions in comparison to reported relapse information, similar to Figure 4. AUCs are listed as the insert. The ROC curves and AUC results also show that pathway-based models are better than gene-based models.

Our earlier results of selected pathway features vs. PAM 50 genes suggested that pathway-based features may be better than gene-based features. To validate this, we trained the four data sets individually and compared within the same data set the performance of pathway-based models and gene-based genomic models which do not have the PDS matrix generation step (Figure 2). In order to test the risk differentiation power of the model, the cutoff PI value in each data set was set to match the ratio of relapse vs. non-relapse patients in that particular set. The results of Kaplan-Meier survival curves and ROC plots based on classification all consistently show that pathway-based genomic models are superior to the gene-based models (Figure 5A–H). For example, in Miller data set the log-rank p-value is 6.25e-12 for the pathway-based model (Figure 5B), compared to that of 1.75e-9 for the gene-based model (Figure 5A). In the Desmedt data set, the p-value of the pathway-based model is even more significant than that of gene-based model (5.12e-36 vs. 8.84e-12, Figure 5H and 5G). Similarly, pathway-based genomic models have better ROC curves than gene-based genomic models (Figure 5I), with AUCs of 0.80 vs. 0.78 in Miller data, 0.85 vs. 0.77 in Pawitan data, 0.74 vs. 0.70 in Ivshina data, and 0.92 vs. 0.76 in Desmedt data. To estimate the statistical significance of comparisons among the pathway-based and gene-based models, we performed leave-one-out cross validation (LOOCV) simulations to compute the Wilcoxon log-rank test p-values and AUCs of ROC classification curves. The cross validation results show that statistically the pathway-based models perform better than the gene-based models (Figure S2, all t-test p-values<0.001). These results are consistent with the observations from previous studies [12],[14], and support the hypothesis that including higher-order secondary information yields better prognostic values.

NKI70 (Mammaprint) is one of the most commonly used model for breast cancer prognosis prediction, and it has been approved by FDA for commercially use in clinics. To demonstrate the potential clinical utilities of our model, we compared the NKI70 method with ours, and applied the NKI70 method to our training data set (Miller data). We first mapped the NKI70 gene signatures [8] to the genes in the U133A array, then correlated the gene-expression profile with the good-prognosis/poor prognosis data from the NKI study and classified the samples into good and poor clusters as done previously [7]. The NKI70 test gives a Wilcoxon log-rank test p-value of 2.58e-3 for the survival analysis, in contrast to the p-value of 6.25e-12 obtained by our pathway-based model; it only yields an AUC of 0.62 for classification, in contrast to 0.80 from our model (Figure S3).

### The Combined Model with Pathway-Based Genomic and Clinical Features Is Superior to the Genomic or Clinical Model Alone

Previous studies suggested that clinical information of breast cancers provides additional values to a genomic model that was built on lists of genes [21]. To test if such merit of clinical information also applies to our genomic model of fifteen pathway features, we investigated the performances of the genomic, clinical and genomic-clinical combined models.

Since the scales of PDS and clinical features vary significantly, we re- normalized PDS and clinical features independently to have the standard normal distribution, so that they are subject to the same selection criteria. The resulting clinical model is composed of four selected features: grade, tumor size, p53 and lymph node. This is not surprising, as they are also significant factors in the univariate Cox-PH models (Table 2 and Figure 1B–E). The combined model keeps ten of the fifteen pathways (Table 2) and about 60% of genes that were selected by the genomic model. It also selects tumor size and lymph node status as additional features (Table 2). This is expected given their highly significant p-values (1.92e-7 and 4.93e-8, respectively) in the univariate Cox-PH models (Figure 1B and 1E), as well as relatively large coefficients in the clinical model (0.27 and 0.36, respectively). Since only testing data set 2 has both tumor size and lymph node information, we used this data set and the testing data set to demonstrate the performances of genomic, clinical, and combined models.

**Table 2:** Selected features in the models.

Features	Coefficients	Hazard Ratio	p-values in univariate COX-PH model
Pathway-based genomic model			
KEGG_MELANOGENESIS*	1.075908	2.93266	0.00188
BIOCARTA_SRCRPTP_PATHWAY*	0.914698	2.49602	1.01e-7
BIOCARTA_AKAP13_PATHWAY*	0.828364	2.28957	0.00351
BIOCARTA_RARRXR_PATHWAY*	0.670795	1.95579	9.58e-6
BIOCARTA_VIP_PATHWAY*	0.635108	1.88723	2.15e-5
KEGG_FATTY_ACID_METABOLISM *	0.520653	1.68313	2.53e-6
BIOCARTA_G1_PATHWAY*	0.520446	1.68278	2.66e-6
KEGG_LINOLEIC_ACID_METABOLISM	0.368615	1.44573	3.55e-4
KEGG_LYSOSOME	0.300587	1.35065	2.2e-6
BIOCARTA_P53_PATHWAY*	0.239062	1.27006	8.74e-4
BIOCARTA_PYK2_PATHWAY*	0.158405	1.17164	1.29e-4
BIOCARTA_GABA_PATHWAY	0.139229	1.14939	0.0162
BIOCARTA_FEEDER_PATHWAY	0.110334	1.11665	0.0218
BIOCARTA_RNA_PATHWAY*	0.037978	1.03871	7.67e-5
BIOCARTA_IL5_PATHWAY	0.012039	1.01211	0.00895
Clinical model			
Lymph Node Status*	0.375874	1.456264	4.46e-7
Tumor Size*	0.270893	1.311135	6.03e-7
Grade	0.126814	1.135206	4.92e-4
P53	0.043517	1.044478	0.0171

*: these pathways and clinical parameters are also selected by the combined model.

**Figure 6:**
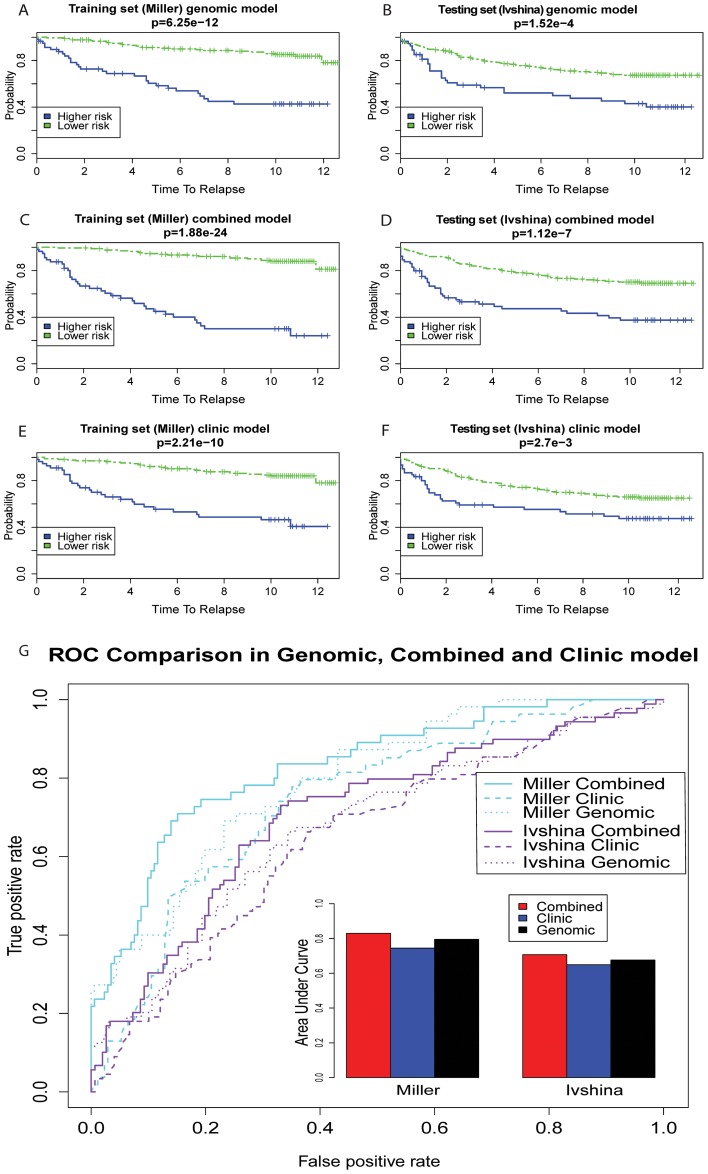
Comparing the prognosis performance from the pathway-based genomic model, the clinical model, and the combined model. Higher risk and lower risk group are determined by the same PI cutoff as in Figure 4. The p-values in Kaplan-Meier curves are calculated using the Wilcoxon log-rank tests. In both the training data set and testing data set 2 (Ivshina data) that have full clinical information, the combined models outperform the pathway-based genomic model, and the pathway-based genomic model outperform the clinical model.

The comparisons present the compelling advantage of combining clinical and genomic information in a model (Figure 6). As shown in the training data, selected clinical features are undoubtedly important: the Wilcoxon log rank test p-value of the clinical model is 2.21e-10 (Figure 6E), slightly less significant than the pathway-based genomic features by two orders of magnitude. Most importantly, the combined model is much better than either genomic model (p-value = 6.25e-12) or clinical model alone, with a p-value of 1.88e-24 (Figure 6C). This trend of significances is consistent in the testing set 2, with the p-values of 1.12e-7 in the combined model (Figure 6D), 1.52e-4 in the genomic model (Figure 6B), and 2.7e-3 in the clinical model (Figure 6F). Moreover, the ROC curve comparisons of these three models also show the same order of performances: combined model>genomic model>clinical model, with AUCs of 0.83, 0.80, and 0.74 in the training set, and 0.71, 0.68 and 0.65 in the testing set 2 (Figure 6G).

To demonstrate the statistical significance of comparisons among the pathway-based, clinical and combined model in the training set and the testing set 2, we performed leave-one-out cross validation (LOOCV) simulations to compute the Wilcoxon log-rank test p-values and AUCs of ROC classification curves. The cross validation results show that statistically the combined model performs better than the pathway-based model, and the pathway-based model performs better than the clinic model (Figure S4, all p-values<0.001 between pathway-base/clinical models and combined models).

### Biological Relevance of Featured Pathways and Genes

We expect that the consensus pathways selected both in our genomic model and combined model convey important cancer-related functions. To test this we examed the annotations of this subset of ten pathways (Table 2). Interestingly, KEGG_MELANOGENESIS is selected as a feature, probably due to inclusion of many cancer relevant genes in this pathway: such as protein kinase genes PRKACB, PRKACG, PRKCB, PRKCA; phosphorylase kinase genes CALM1, CALM2, CALM3; G-protein related gene GNAQ, HRAS; mitogen-activated protein kinases MAPK1, MAPK3, MAP2K1; and other oncogenes like RAS [31],[32]. Many of these genes have been shown to function in breast cancer progression [31]. Impressively, multiple signaling pathways are selected, including BIOCARTA_P53_PATHWAY, BIOCARTA_SRCRPTP_PATHWAY, BIOCARTA_PYK2_PATHWAY, BIOCARTA_VIP_PATHWAY, BIOCARTA_RARRXR_PATHWAY, and BIOCARTA_AKAP13_PATHWAY. They are well-known to be associated with breast cancers prognosis [33]–[39]. The best example is BIOCARTA_P53_PATHWAY, the dysregulation of p53 Signaling Pathway is well-documented, and the tumor-suppressor gene p53 has one of the highest mutation rates in breast cancer [5],[19].

In addition, some pathways related to basic cell functions are selected as prognostic features. For example, G1_PATHWAY is selected, and the G1/S cell cycle checkpoint controls are well known to be dysfunctional in many cancers including breast cancer [40]. FATTY_ACID_METABOLISM is also selected by the model, and many studies have showed that fatty acid metabolism is involved in breast cancer [41]. In particular, Fatty acid synthase (FASN) is highly expressed in breast cancer with a poor prognosis compared to others [41]. Interestingly, BIOCARTA_RNA_PATHWAY is also selected, largely due to its members TP53 and MAP3K14 that are closely related to breast cancer.

A total of 265 genes are overlapped between the selected pathways of the genomic model and the combined model. Table 3 summarizes the top 30 genes that are involved in the selected pathways. They are ranked by weighted sum of both occurrences in selected pathways (counts) and weights measured by the hazard ratio of each pathway. Among them, many genes encode protein kinases that are well-known to be involved in breast cancers, such as PRKACB, PRKACG, MAPK1 and CALM1. Some other genes encode transcription factors that are well-known for their close relationship to cancer, such TP53, RB1, HRAS, RAF1, GRB2, E2F1, and SRC [32],[42]–[44]. We therefore conclude that the selected pathways are prognostic features of significant cancer relevance.

**Table 3:** Top 30 most frequent genes in the pathways of the genomic model and the combined model.

Gene ID	Genomic Model Counts	Combined Model Counts	Weighted Genomic Model Counts*	Weighted Combined Model Counts*
PRKACB	3	4	7.109452	4.46549683
PRKACG	3	4	7.109452	4.46549683
PRKCB	3	6	6.600318	6.54794229
PRKCA	3	6	6.600318	6.54794229
CALM1	3	6	5.991523	6.32738762
CALM2	3	6	5.991523	6.32738762
CALM3	3	6	5.991523	6.32738762
GNAQ	3	4	5.991523	4.26250968
SRC	3	5	4.817049	5.3291015
GSK3B	2	3	4.615435	3.23989145
CDC25A	2	2	4.178799	2.25477095
CDK1	2	2	4.178799	2.25477095
PRKAR2A	2	3	4.176796	3.26764027
PRKAR2B	2	3	4.176796	3.26764027
HRAS	2	4	4.104297	4.27393317
MAP2K1	2	4	4.104297	4.27393317
MAPK1	2	4	4.104297	4.27393317
MAPK3	2	4	4.104297	4.27393317
RAF1	2	4	4.104297	4.27393317
MAP2K2	2	4	4.104297	4.27393317
TP53	3	3	3.991545	3.03875447
GRB2	2	4	3.667662	4.32604023
CYCSP35	2	5	3.058867	5.12953106
PLCG1	2	4	3.058867	4.13411496
MAP3K1	2	3	3.058867	3.06465312
E2F1	2	2	2.952836	2.0232666
RB1	2	2	2.952836	2.0232666
CCND1	2	2	2.952836	2.0232666
CDK4	2	2	2.952836	2.0232666
CCNE1	2	2	2.952836	2.0232666

## Discussion

The heterogeneity of cancers is being increasingly recognized, suggesting more personalized care decisions with treatment for individual patients are needed. As a result, prognosis prediction of breast cancers with high-throughput data has been a growing topic in recent years. Many statistical and machine learning methods have been developed to analyze various types of high-throughput cancer genomics data, by taking advantage of higher-order relationships among genes. The hypothesis is that the highly correlated gene-based markers often represent identical biological processes; therefore by including higher-order representative features, such as Gene Ontology sets, pathways and network modules, the prediction will be more stable [9]–[14],[45]. Our novel method of prognosis prediction presented in this study belongs to this class of methods. However, unlike some other methods where individual pathway information is lost due to summarization or transformation, the pathway features proposed in this study explicitly measure the degrees of pathway dysregulation for cancer recurrence. Comparing selected pathways and the PAM50 genes which were demonstrated to be prognostic [20], the PDS-based pathway approach has better correlation to breast cancer relapses. Moreover, when comparing gene-based with the pathway-based genomic models, where the only difference between them was the input matrix, pathway-based models uniformly performed better than gene-based models in all the data sets we tested. Our results are consistent with several other gene-set/pathway-based models [9],[14], where different summarization metrics were used. It will be very interesting to compare the prediction results based on these different metrics in a follow-up study.

To demonstrate the robustness in predicting differential risks of relapse from the pathway-based genomic model, we chose to train and test on independent study samples, rather than combining them together as a large data set [21],[46], which would diminish the effect of population heterogeneity. Despite population difference and much bigger testing data size relative to the training data size, the method still achieved good performance on all three testing data sets, including a data set of all early stage lymph node negative tumors where prognosis is particularly difficult to predict. Another merit of our method is that it enables combining the important clinical information with the pathway-based genomic information. Even though the clinical model by itself is the least predictive, compared to the genomic model and the combined model, it is nevertheless significant and informative, as shown by tumor size and lymph node status. The genomic model is better than clinical model alone. However, the combined model of clinical and genomic features performs the best. Our conclusions agree and extend the earlier work from Fan et al. [21] who focused on prognosis prediction of all node-negative and systemically untreated breast cancer patients, since we include both node-negative and node-positive samples. The results of the genomic model (AUC = 0.80 and p-value = 6.25e-12 in training data, and AUC = 0.68 and p-value = 1.52e-4 in test data 2) and the combined model (AUC = 0.83 and p-value = 1.88e-24 in the training set, and AUC = 0.79 and p-value = 1.12e-7 in test data set 2) are better than what was recently reported by Vilinia S et al [47]. They obtained an AUC = 0.74 for the training set and 0.65 for the testing set, in a model that combined signatures of mRNA and microRNAs deriving from the TCGA IDC cohort sequencing data. This suggests the advantages of combining PDS based pathway score inputs with a Cox-PH model and LASSO penalization approach: even though the genomic data in our study are based on microarrays that have more noise and smaller sample sizes, they still yield better predictive results in comparison to the combined mRNA and microRNA sequencing signatures obtained from a larger sample size. It will be of great interest to apply our models to the TCGA breast cancer mRNA and microRNA sequencing data in the future.

The pathways selected by the model show biological relevance to breast cancer prognosis. The fatty acid metabolism pathway is found to be crucial to maintain the cancer cell malignant phenotype, and higher expression of fatty acid synthase has been discovered as a common phenotype in breast cancer with a poorer prognosis [41]; As another example, Src kinase activation by protein tyrosine phosphatase alpha (SRCRPTP_PATHWAY), has been discovered in invasive breast cancer with compelling evidences. Src inhibitors are being considered as potential therapy to treat invasive breast cancers, as inhibition of c-src was recently found to be involved in E2-induced stress which would finally result in apoptosis in breast cancer cells [33]. Increasing evidence shows that vasoactive intestinal peptide (VIP) in BIOCARTA_VIP_PATHWAY is highly expressed in breast cancer cells along with its receptor [33], and VIP-targeted nanomedicine is under study as therapy for breast cancer [34]. Pyk2 in BIOCARTA_PYK2_PATHWAY is linked to map kinases MAPK, which has wealthy records in breast cancer studies [35]. RARRXR_PATHWAY is the RAR/RAR nuclear receptor complex that is co-activators to facilitate initiation of transcription in carcinoma cells [37]. And BRX, the truncated form of Rho-Selective Guanine Exchange Factor AKAP13 in the BIOCARTA_AKAP13_PATHWAY, has been identified to function as an ER cofactor [39].

Although the workflow proposed in this study is generic and the pathway features are clearly significant, we should point out a few potential limitations of the model. First of all, the pathway-based model is trained and tested on gene expression data from the U133A platform. We suspect that direct application of the model to other platforms, such as RNA-Seq, is not desirable, and some additional re-processing work has to be done additionally. The reason is that data distributions maybe very different between various platforms. One notorious example is that biomarkers identified by high-throughput microarray platform often had poor correlations in qPCR platform. Thus we recommend that when researchers use the workflow in Figure 2 on different data types, they may increase the predictive power by retraining the model with their own data. Another limit of our approach is that we only used the information from genes that compose the 403 pathways that we considered, thus some gene-level information is unavoidably lost. In our case, over 4500 genes were enlisted in the pathways, and among them over 3200 genes are probably expressed (averaged log 2 expression intensities >7). On the other hand, the raw U133A array has results of over 14,000 genes within which over 10,000 genes are probably expressed. Therefore our model captures about 1/3 of the gene-level information overall. One can certainly use other curated gene sets, such as the MsigDB C2 gene sets, to increase the coverage of the genes by the pathways. However, from the sensitivity analysis that we have performed (Figure S1), we only observed a slight decrease of model performance based on AUCs, which are in the range of 0.69 and 0.81.

In conclusion, we propose a novel pathway-based genomic model that measures the pathway-based deregulation score and shows significant prognosis values. This pathway-based genomic model performs better than the gene-based genomic model. Additionally, we found that combining the clinical information of lymph node status and tumor size improves the performance of the prognosis model. Many selected pathways in our study present values for breast cancer prognosis prediction, and they are also promising therapeutic targets for future investigations.

## Materials and Methods

### Study Population

We used four publicly available data sets of breast cancer samples from National Center for Biotechnology Information (NCBI) Gene Expression Omnibus (GEO) GSE4922 [22], GSE1456 [23], GSE3494 [19] and GSE7390 [24]. All four data sets are based on Affymetrix HG-U133A microarray platform, and have relapse-free survival information as well as some other clinical information, as shown in Table 1. For data set GSE7390 [24], all patients are lymph node negative. The GSE3494 data set was used as the training set as it has more clinical information, and all others were used as testing data sets.

### Microarray Gene Expression Data Processing and Analysis

We mapped original probe IDs to Gene IDs using R package *biomaRt* [48]. In order to relate the probe ID to the Gene ID, we downloaded the array annotation file and used the RefSeq IDs as the intermediates to map to the Gene ID. When a gene has multiple probes, we computed the geometric mean of log2 transformed probe intensities as the gene expression. All the data sets were normalized independently between array using *limma* package [49]. To minimize batch effects across different data sets, we used the CONOR package with the Bayesian method [50].

We generated the PAM50 heatmap of the gene expression data and the correlation heatmap with hierarchical clustering, where Euclidean distance measure was employed. For the clinical factors, we correlated their associations with the relapse in the training data set with both Chi-square test and Wilcoxon log-rank test for survival curves.

### Prognostic Pathway-Based Classifier Selection

The pathway information was obtained from the GSEA (http://www.broadinstitute.org/gsea/) curated gene sets that include a total of 403 pathways from Biocarta (http://www.biocarta.com) [51]and KEGG [52]. To perform gene sets analysis, we used R package *Pathifier* [25], an algorithm that transforms the information from the gene level to pathway level and infers pathway deregulation scores for each pathway within each sample. The pathway deregulation score (PDS) in each sample is a measure of degrees of the deviation of a specific pathway from the “normal status” located on the principle curve. The concept of principle curve was proposed by Hastie and Stuetzle [53] as a nonparametric nonlinear extension of the PCA (Principle Component Analysis) in which the assumptions of dependence in the data are avoided. A principle curve is a one-dimensional curve that is derived from the local average of p-dimensional points and goes through the cluster of p-dimensional principle components. It sensibly captures the information of variation in all the samples. Specifically, the single parameter *λ* varies tracing the whole data along the curve [53]. The curve *f*(*λ*) is defined to be a principal curve if 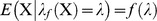 for arbitrary *λ*. The principle curve is built through iterations of smoothed procedure in the local average of data points. If one sample differs from others in one specific pathway, the distance to the curve is further and it leads to a higher PDS score and vice versa.

In the model selection stage, we used Cox-Proportional Hazards (Cox-PH) model based on L1 – penalized (LASSO) estimation [27]–[29], with the R package *penalized* [29]. With the input of both PDS score containing the gene sets information and survival information of time and relapse, a tuning parameter lambda was used to restrict the number of parameters in the model. The optimal lambda was selected after running 250 simulations through likelihood cross-validation. A prognostic genomic model was thus generated with specific pathways and coefficients. We then computed a Prognosis Index (PI) score which is the logarithm of hazard ratio. We divided the samples into two groups of higher risk and lower risk with a 3 to 1 ratio, based on the 3rd quartile of PI. We used this cutoff to reflect the relapse/non-relapse ratio in the training data set.

We tested the above model in three other data sets. To do so we used the same PI cutoff above and separated samples into predicted high risk and low risk groups. We then used Kaplan-Meier curve together with Wilcoxon log rank test to evaluate the performance of our model. To generate the receiver operating characteristic (ROC) curves, PIs are used as predicted values in comparison to the “truth” values of relapse/non-relapse information. The confusion matrix with sensitivity and 1- specificity is calculated for each division in ROC curves and the areas under the curve (AUC) is shown along with the ROC plot.

### Combined Molecular and Clinical Model

To determine whether the clinical factors improve the prognosis of genomic pathway-based model, we re-normalize the clinical factors and molecular PDS independently to ensure that each factor has the standard normal distribution. We then combined the normalized clinical and molecular factors into the LASSO penalized step and built the combined model using the optimized lambda through 250 simulations, similar to the construction of the genomic model as described earlier. The model performance comparisons were also done similarly to those of the genomic model.

### Survival Analysis

We used survival analysis to compare the relapse-free-survival results in the training and testing data sets. Patients without these events during the study were considered censored. We used the Cox-PH model to associate the risk of relapse to selected pathway features and clinical features by L1- LASSO. The Cox model is a semi-parametric model that is widely used to analyze the survival data. The non-parametric portion comes from the fact that no assumptions are made about the form of the baseline hazard. However, it has the assumption that the log hazard ratios are constant over the time for each feature. Assume that we obtained *p* features to be related with breast cancer relapse for each patient 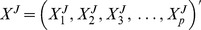, Cox-PH model represents the relationship between the risk of relapse and *X* features as:
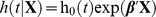
Here 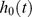 is the baseline hazard (instantaneous risk) which only depends on time. The ratio of hazard (HR) between two pathway or clinical features 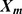 and 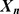 is:
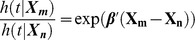
The relative hazard between any two features is constant over time and only depends on the differences of the values in features. The PI for each patient J's features is calculated as
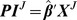
This risk factor can be easily transformed to hazard ratio for different features, assuming that we have a baseline feature. The weights 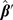 for different features were calculated from the training data set using the Cox-LASSO model.

For the genomic, clinical and combined models, we used Kaplan Meier curves to present the prognosis performance in classified high risk and low risk groups. The data set was dichotomized into two groups, and the higher risk group is assumed to have higher hazard of relapse compared to the lower risk group. We used the Wilcoxon log-rank test to check the survival difference between these two groups. To find the significance of an individual factor's impact on relapse, we fit individual predictor with a univariate Cox-PH model. We then calculated the hazard ratio by computing the exponential of the coefficients in the Cox-PH model. All survival analysis was conducted using the R package *Survival* [54].

### Sensitivity Analysis of Pathway-Based Models

To examine the effect of input pathways on model performance, We randomly select 1/2, 1/4, 1/8 and 1/16 of all input KEGG and BioCarta pathways, then generated the PDS Matrices for 18 times under each case. For each simulation, we built the model with the workflow in Figure 2 and computed the Wilcoxon log-rank test p-value between the survival curves of two risk groups, as well as the AUC of the classification results. We then used boxplots to demonstrate the differences of –log10 (p-values) and AUCs due to different total pathway counts.

To estimate the statistical confidence of comparisons of each model, we used leave one out cross validation (LOOCV) to compute p-values and AUCs across all simulations. In the *i*th simulation (*i* = 1,…,total sample size of the data set), we deleted the *i*th patient sample, modified the PI threshold by the remaining sample ratio of recurrence to non-recurrence and finally calculated the Wilcoxon log-rank test p-value as well as the AUC of the classification results. We then used boxplots to demonstrate the comparisons between the pathway-based and the gene-based models, and among the genomic, clinical, and the combined models.

### Comparison to the NKI70 Model

We tested the NKI70 method to our training data set (Miller data). We mapped the NKI70 gene signatures from to the genes in the U133A array. We correlated the gene-expression profile with the good-prognosis/poor prognosis data from the NKI study [8], and then classified the samples into good and poor clusters as done by others [7]. For consistency, we used the Wilcoxon log-rank test p-value from survival analysis and the AUC of the ROC classification to assess the results.

## Supporting Information

Figure S1 **The effect of removing pathways on model performance (both P-values and AUCs).** A fraction (1/2, 1/4, 1/8 and 1/16) of the initial 403 pathways are randomly selected to generate PDS matrices over 18 simulations, followed by the flowchart in Figure 2. Boxplots of AUCs from ROC curves are shown.
(TIF)

Figure S2 **Cross validation results to compare the pathway-based and gene-based models on the 4 data sets in** **Figure 5** **.** Leave-one-out cross validation (LOOCV) was performed to compute the Wilcoxon log-rank test p-values (A) and AUCs (B) across all simulations. All pairs have t-test p-values<0.001.
(TIF)

Figure S3 **Comparison of ROC performance between the NKI70 method and our method on Miller dataset.**
(TIF)

Figure S4 **Cross validation results to compare the genomic, clinical, and combined models on the 2 data sets in** **Figure 6** **.** Leave-one-out cross validation (LOOCV) was performed to compute the Wilcoxon log-rank test p-values (A) and AUCs (B) across all simulations. All pairs have t-test p-values<0.001.
(TIF)
