# Supplementary figures and images for: A Novel Model to Combine Clinical and Pathway-Based Transcriptomic Information for the Prognosis Prediction of Breast Cancer
Source: PLoS Comput Biol. 2014 Sep 18;10(9):e1003851. doi: 10.1371/journal.pcbi.1003851 (PMC4168973; doi:10.1371/journal.pcbi.1003851)

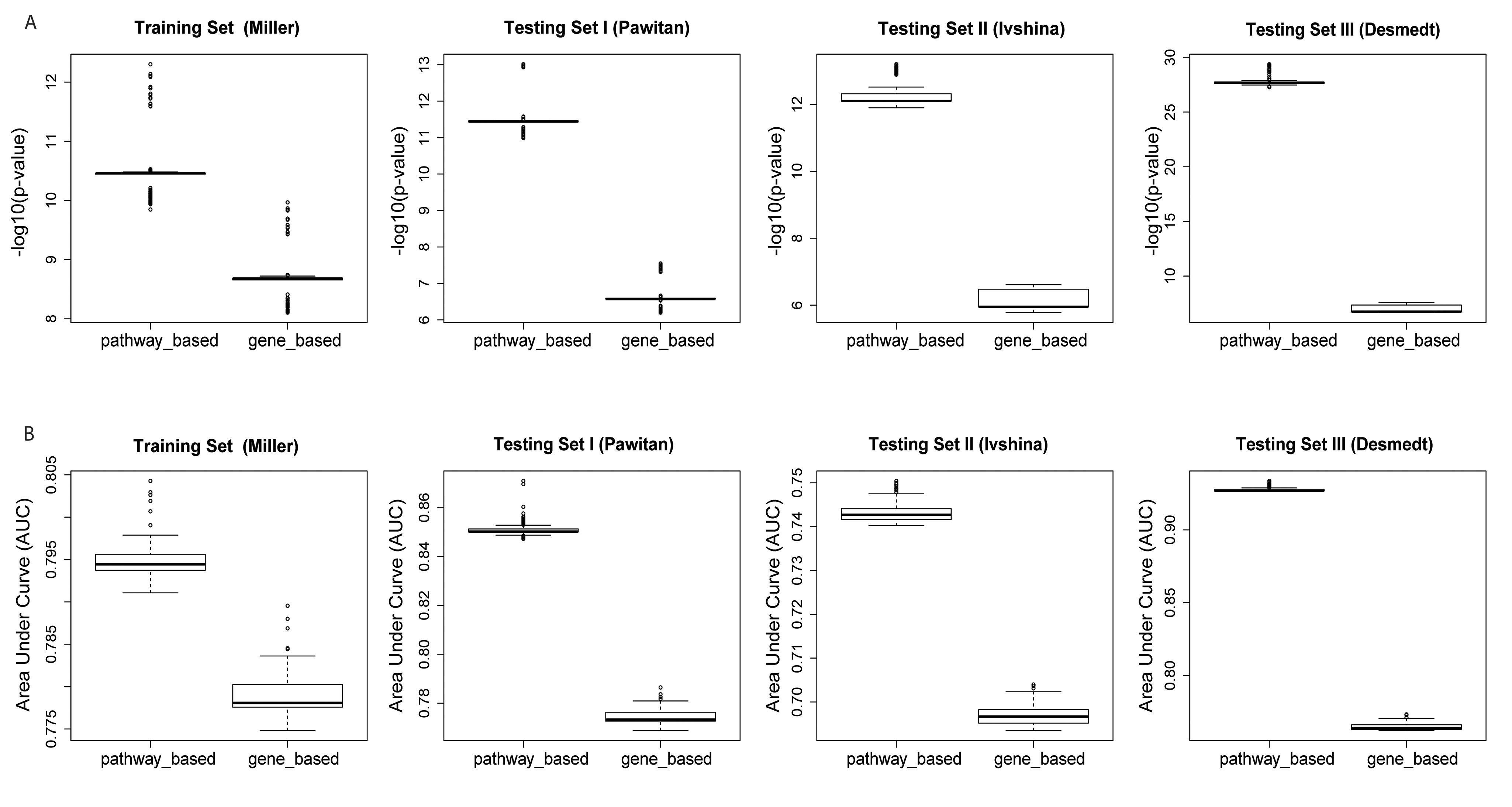

Supplement: Figure S2 — Cross validation results to compare the pathway-based and gene-based models on the 4 data sets in Figure 5 . Leave-one-out cross validation (LOOCV) was performed to compute the Wilcoxon log-rank test p-values (A) and AUCs (B) across all simulations. All pairs have t-test p-values<0.001. (TIF) [file pcbi.1003851.s002.tif]

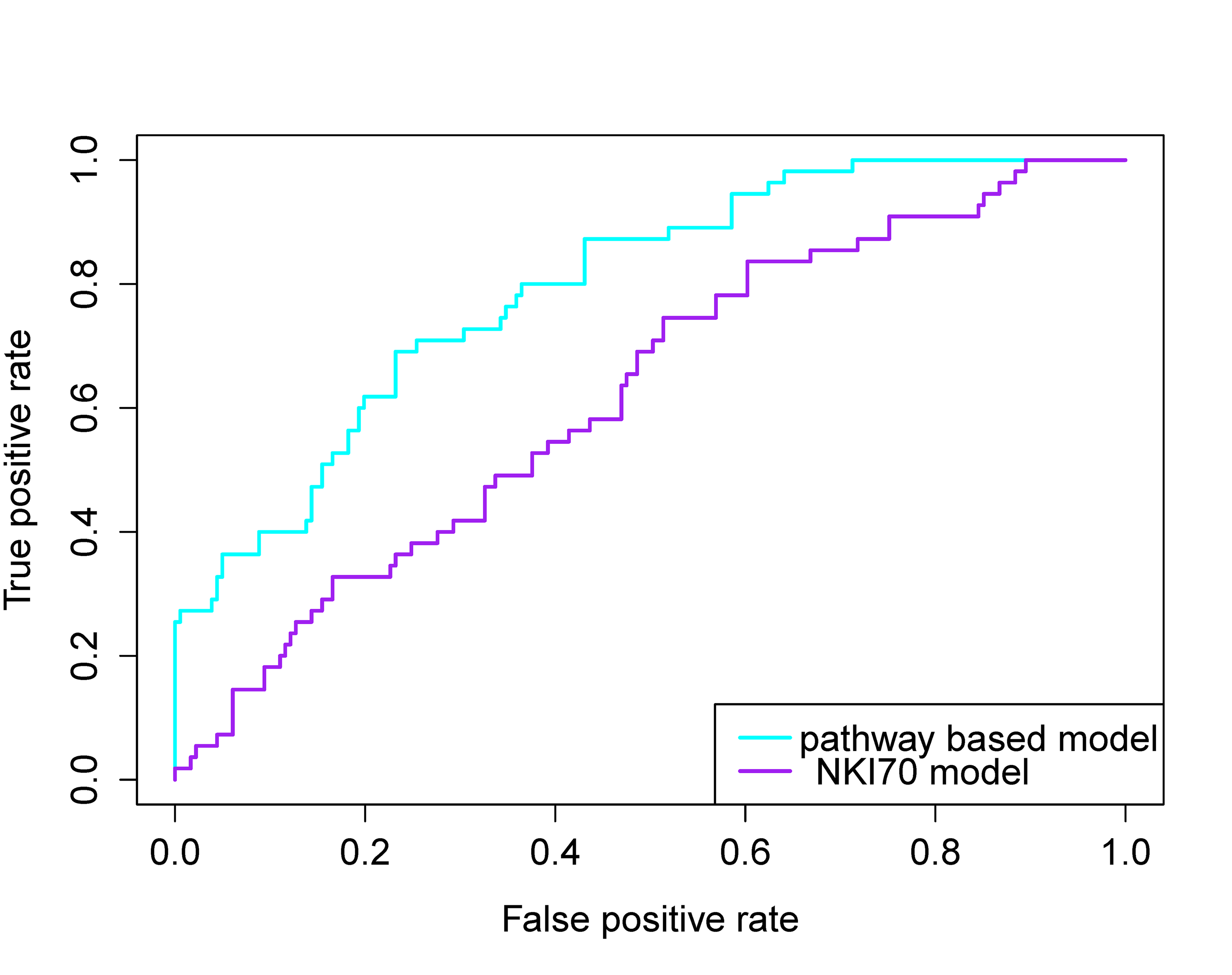

Supplement: Figure S3 — Comparison of ROC performance between the NKI70 method and our method on Miller dataset. (TIF) [file pcbi.1003851.s003.tif]
